# Epigenetic Regulation of Bovine Spermatogenic Cell-Specific Gene *Boule*

**DOI:** 10.1371/journal.pone.0128250

**Published:** 2015-06-01

**Authors:** Wang Yao, Yinxia Li, Bojiang Li, Hua Luo, Hongtao Xu, Zengxiang Pan, Zhuang Xie, Qifa Li

**Affiliations:** 1 College of Animal Science and Technology, Nanjing Agricultural University, Nanjing 210095, China; 2 Institute of Animal Science, Jiangsu Academy of Agricultural Sciences, Nanjing 210014, China; University Hospital of Münster, GERMANY

## Abstract

Non-primate mammals have two deleted azoospermia (DAZ) family genes, *DAZL* and *Boule*; genes in this family encode RNA-binding proteins essential for male fertility in diverse animals. Testicular *DAZL* transcription is regulated by epigenetic factors such as DNA methylation. However, nothing is known about the epigenetic regulation of *Boule*. Here, we explored the role of DNA methylation in the regulation of the bovine *Boule* (b*Boule*) gene. We found that a long CpG island (CGI) in the b*Boule* promoter was hypermethylated in the testes of cattle-yak hybrids with low b*Boule* expression, whereas cattle had relatively low methylation levels (*P* < 0.01), and there was no difference in the methylation level in the short CGI of the gene body between cattle and cattle-yak hybrids (*P* > 0.05). We identified a 107 bp proximal core promoter region of b*Boule*. Intriguingly, the differences in the methylation level between cattle and cattle-yak hybrids were larger in the core promoter than outside the core promoter. An *in vitro* methylation assay showed that the core promoter activity of b*Boule* decreased significantly after M.SssI methylase treatment (*P* < 0.01). We also observed dramatically increased b*Boule* transcription in bovine mammary epithelial cells (BMECs) after treatment with the methyltransferase inhibitor 5-Aza-dC. Taken together, our results establish that methylation status of the core promoter might be involved in testicular b*Boule* transcription, and may provide new insight into the epigenetic regulation of *DAZ* family genes and clinical insights regarding male infertility.

## Introduction

Spermatogenesis is an extremely complex process of cell differentiation, and includes three specific functional phases: spermatogonia proliferation, spermatocyte meiosis, and spermatid differentiation. Spermatocyte meiosis is a key step in spermatogenesis, and defects in genes controlling spermatocyte meiosis, such as microdeletions, mutations, and decreased expression, lead to meiotic arrest, impaired spermatogenesis, and male infertility [1–4]. The deleted in azoospermia (*DAZ*) gene family is distinctly involved in meiosis during spermatogenesis, and consists of three members, *DAZ*, *DAZL* (*DAZ*-Like), and *Boule* [5–7]. *Boule* is the recently identified ancestral “grandfather” gene in the *DAZ* family; it is expressed in prophase and metaphase spermatocyte meiosis in the testis, and highly expressed in meiotic pachytene spermatocytes [5]. *Boule* is found in vertebrates and invertebrates [5, 8–10]. *DAZL* is regarded as the “father” gene in the *DAZ* family and evolved from ancestral *Boule* [5]. It is expressed in spermatogonia and spermatocytes of the testis and ovary [11]. *DAZL* is only detected in vertebrates [7, 10–12]. *DAZ* maps to the Y chromosome, is obtained by gene transposition, duplication, and exon splicing from autosomal *DAZL*, and is highly expressed in meiotic prophase germ cells in the testis. *DAZ* is only found in Old World monkeys and humans [13–15]. The proteins encoded by DAZ family genes are all RNA-binding proteins with typical RNA-recognition motifs (RRM) and DAZ repeats; they play an important role in spermatocyte meiosis and are associated with male infertility [5–8, 16–17].

*Boule*, a recently identified member of the *DAZ* family, was first detected in *Drosophila* and human testes [5, 18]. In *Drosophila*, the testes of *boule* mutants produce no sperm and have germ cells that are arrested before meiosis, resulting in azoospermia and male infertility [19]. A fly *boule* transgene or a human *BOULE* transgene can rescue the reproductive defects of *boule* mutant flies [18, 19]. Testicular BOULE expression is decreased in some patients with abnormal spermatogenesis, and spermatogenesis is arrested before the primary spermatocyte stage; no BOULE expression is detected in testes of patients with complete meiotic arrest [20]. Lin et al. [21] also found that *BOULE* mRNA levels are significantly decreased in azoospermic male testes, and are progressively decreased with increasing severity of testicular failure; patients with successful sperm retrieval have significantly higher *BOULE* levels than patients with failed sperm retrieval. *Boule* ^−/−^ mice are male sterile and azoospermic [22], similar to *boule* mutant flies and some men with DAZ deletions [13, 18]. Li et al. [23] demonstrated that over-expression of *Boule* promotes the expression of meiosis-related genes such as *Stra8* in goat male germline stem cells. Thus, these results suggest that the expression of *Boule* is associated with mammalian spermatocyte meiosis and male infertility, and that it may be the key regulatory factor of spermatocyte meiosis.

The transcriptional regulation of *DAZ* family genes has been extensively studied [24–31]. However, little is known about the regulation of *Boule* [3, 23, 32], and particularly its epigenetic regulation. Our previous study suggested that bovine *Boule* (b*Boule*) may function in bovine spermatogenesis, and that low b*Boule* expression might lead to male sterility in cattle-yak hybrids [8, 33]. In the present study, we examined the epigenetic mechanisms of low b*Boule* expression in testes of cattle-yak hybrids.

## Materials and Methods

### Bioinformatic analysis

The genomic DNA sequence of the b*Boule* gene was obtained by a BLAST search of the genome database of cattle (*Bos taurus*) (http://www.ncbi.nlm.nih.gov/genome/82) based on the cDNA sequence of b*Boule* (GenBank ID: EU050657) that was previously cloned by our group [8]. The putative promoter region of b*Boule* was predicted using Proscan software (http://www-bimas.cit.nih.gov/molbio/proscan/). CpG islands (CGI) were searched by the online CpG Island Searcher program (http://ccnt.hsc.usc.edu/cpgislands2/cpg.aspx). We searched the transcription factor binding sites (TFBS) of the b*Boule* core promoter using the web tool TFSEARCH v1.3 (http://www.cbrc.jp/research/db/TFSEARCH.html) using a threshold score of 85.0.

### PCR and sequencing

Genomic DNA was isolated from testes using the phenol-chloroform method. Three primers for the amplification of the b*Boule* promoter region were designed by Primer Premier 5.0 software based on the genomic DNA sequence of b*Boule* (**Table 1**). The reaction mixture and PCR program were described in Luo et al. [34]. PCR products were separated using 1.2% agarose gel electrophoresis, purified using a DNA Purification Kit (Axygen, Union City, CA, USA), and sequenced by Invitrogen (Shanghai, China).

**Table 1:** The primers used in this study.

	Gene	GenBank ID	Primer sequence (5'-3')	Size/bp	Tm/°C	Usage
P1	b*Boule*	NW_001494657	F: TTTAGATCTCTCGATCCGCTCACCTCA R: GGGAATCTTCACCCAGGAAGCAACACC	297	60.5	vector construction
P2	b*Boule*	NW_001494657	F: TTTAGATCTACGGGCCACGACCGAAACCT R: GGGAATCTTCACCCAGGAAGCAACACC	224	59.8	vector construction
P3	b*Boule*	NW_001494657	F: TTTAGATCTAGGTTCAGGCCCTGGGTT R: GGGAATCTTCACCCAGGAAGCAACACC	107	56.7	vector construction
P4	b*Boule*	NW_001494657	F: GAGAGTGGTTTGAGAATAGAGTATT R: TTCACACCCAAAAAACAACA	324	50.6	BSP for long CGI
P5	b*Boule*	NW_001494657	F: GAGGGAGGGATGTTGTAAATAA R: TAATTTTAAAAAAATATTATTT	444	56.0	BSP for short CGI
P6	b*Boule*	NM_001102115	F:CAAGTGCCATTGCTATGCCTGC R: GGTTCATTGAAGCTGGATCTCGG	157	60.0	qRT-PCR
P7	*β-actin*	NM_173979	F:TCCAGCCTTCCTTCCTGGGCAT R: GGACAGCACCGTGTTGGCGTAGA	116	56.0	qRT-PCR

BSP, bisulfite sequencing PCR. CGI, CpG island.

### BSP methylation analysis

The testes were collected from healthy adult cattle (male, n = 8) and cattle-yak hybrids (male, n = 8) provided by the Songpan Bovine Breeding Farm (Sichuan, China), and frozen in liquid nitrogen immediately. All animal work was approved by the Animal Ethics Committee at Nanjing Agricultural University. Extraction and bisulfite conversion of genomic DNA and bisulfite sequencing PCR (BSP) were performed according to the methods described by Luo et al. [34]. Primers for BSP were designed by Methyl Primer Express v1.0 software, and are shown in **Table 1**.

### Deletion construction

To create the deletion constructs, we designed three pairs of primers (P1–P3, **Table 1**) for the amplification of three successively shorter PCR products, which were 107 bp (-172/-66), 224 bp (-289/-66), and 297 bp (-362/-66) in length. All primers used had the *Hin*dIII endonuclease site incorporated at the 5′ end and the *Bgl*II site at the 3′ end, and the downstream primers were all the same. PCR products were subcloned into the pGL3 luciferase reporter vector (Promega, Madison, WI, USA) with *Hin*dIII/*Bgl*II sites, and transformed into *Escherichia coli* to generate the luciferase reporter plasmid. Recombinant plasmids were verified by sequencing and named pbBoule-107, pbBoule-224, and pbBoule-297.

### Cell lines and cell culture

Mouse spermatogonia cell line GC-1 (ATCC CRL-2053) and African Green Monkey SV40-transformed kidney fibroblast cell line COS-7 (ATCC CRL-1651) were cultured in Dulbecco's modified Eagle's medium (DMEM) with high glucose, supplemented with 10% (v/v) fetal bovine serum (FBS), 100 U/mL penicillin G, and 100 μg/mL of streptomycin sulfate in a 5% CO_2_ incubator at 37°C.

### Transfection and luciferase assay

The cells were seeded in 48-well culture plates, and transfected with 1 μg/well of promoter-reporter plasmids or empty vector along with 10 ng/well of Renilla luciferase expression vector pRT-TK as an internal control using Lipofectamine 2000 reagent (Invitrogen). After 24 h, luciferase activity was measured using the Dual-Luciferase Reporter Assay Kit (Promega) with a Modulus Single Tube Multimode Reader (Turner Biosystems, Sunnyvale, CA, USA) according to the manufacturer's protocol. Results are expressed as Renilla/firefly luciferase activities.

### M.SssI treatments

The core promoter fragment of b*Boule* was methylated with 2 μL of M.SssI methylase (NEB, Ipswich, MA, USA) at 37°C for 16 h. The completion of the methylation reaction was confirmed by digestion of the fragment with methylation-sensitive *Hpa*II restriction endonucleases (NEB), which cannot cleave DNA if their cognate restriction sites are methylated. The methylated core promoter fragment was then ligated to the same sites of the pGL3 vector (Promega), and transfected into GC-1 and COS-7 cells. Luciferase assays were performed 36 h after transfection.

### 5-Aza-dC treatments

Bovine mammary epithelial cells (BMECs) that do not express b*Boule* were isolated from the mammary tissues of Holstein cows collected during lactation. Cells were seeded in 96-well plates and grown to 80% confluence, then treated with various concentrations (0, 0.05, and 0.5 μmol/L) of fresh 5-aza-2′-deoxycytidine (5-Aza-dC) (Sigma, St. Louis, MO, USA) for 48 h. After 48 h with or without 5-Aza-dC, cells were washed twice with phosphate-buffered saline and harvested. Total RNA was isolated, and the mRNA levels of b*Boule* were measured by qRT-PCR with P6 primers (**Table 1**) according to the ΔΔC_T_ method; *β-actin* was used as the internal control for normalization.

### Statistical analysis

All data are expressed as means ± SEM. The statistical analysis was performed using SPSS v11.0 software (SPSS Inc., Chicago, IL, USA). A two-tailed Student’s *t*-test and ANOVA were used to evaluate the statistical significance of the differences in our experiment data, and Duncan's multiple comparisons test was used for ANOVA. A value of *P* < 0.05 was considered statistically significant.

## Results

### Differential methylation of testicular b*Boule* promoter CGI between cattle and cattle-yak

We detected two CGIs within the 70 kb genome sequence of b*Boule* consists of a 3 kb of the 5' proximal flanking region and a 2 kb of the 3' proximal flanking region. The long CGI was located between nt -2,074 and nt +225 (2229 bp), and included the 5' proximal flanking region, exon 1, and intron 1, with an observed/expected ratio of 0.807 and C+G content of 60.6%. The short CGI was located from nt +20,565 to nt +21,348 (784 bp) in intron 5, with an observed/expected ratio of 0.719 and C+G content of 60.2%.

**Fig 1:**
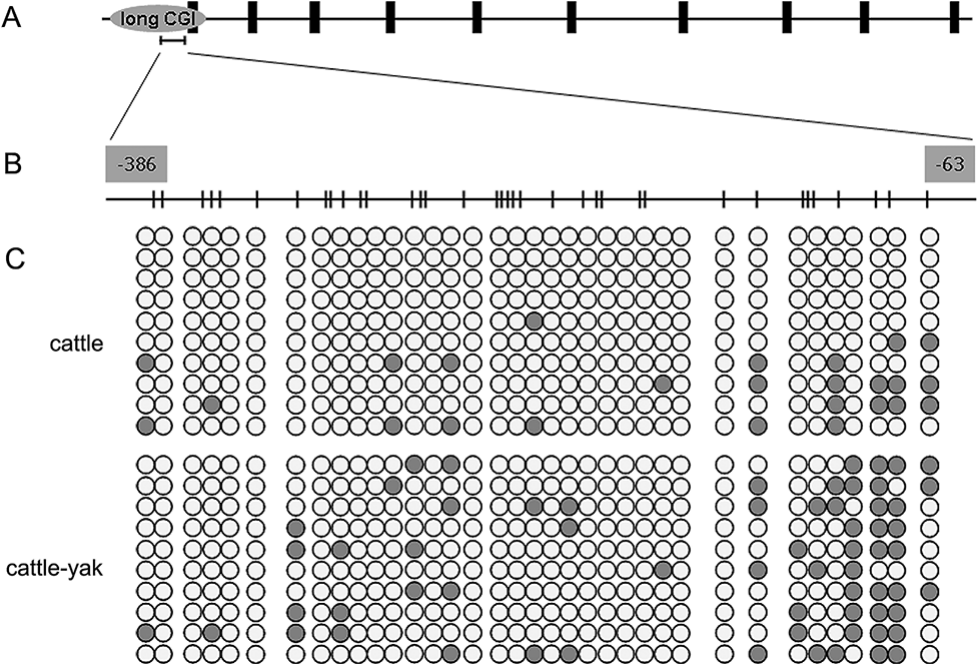
The methylation profile of the long CpG island in the b*Boule* 5' flanking region. (A) Schematic diagram of the long CGI within the b*Boule* promoter. (B) Schematic depiction of the CpG sites for methylation analysis. Nucleotide numbering is relative to +1 at the initiating ATG codon. The short vertical bars represent the CpG dinucleotides. (C) Methylation status of the b*Boule* promoter in the testes of cattle and cattle-yak hybrids. Each line represents an individual bacterial clone that was sequenced. Open circles indicate unmethylated CpG sites. Black circles indicate methylated CpG sites.

At present, studies of the regulation of gene expression by methylation mainly focus on promoter CGI regions [34–36]. Our previous study demonstrated that b*Boule* is expressed at low levels in testes of cattle-yak hybrids with male sterility [8]. To examine whether low b*Boule* expression was associated with methylation in promoter CGIs, we first determined the methylation status of the promoter CGIs (**Fig 1A and 1B**) by BSP using genomic DNA isolated from cattle-yak testes (the males have meiotic arrest and are sterile) and their male parent cattle (with normal meiosis and spermatogenesis). An analysis of the long CGI within the promoter region revealed differences in the methylation profile of the CpG sites between testicular tissue samples of the two bovine populations (**Fig 1C**). The methylation level of the long CGI in cattle-yak testes with male sterility (17.78%, 64/360) was significantly higher than in cattle (6.94%, 25/360) (*P* < 0.01). These data indicate that hypomethylation of promoter CGIs may be associated with low b*Boule* expression in cattle-yak testes.

#### Similar methylation profiles for cattle and cattle-yak testicular b*Boule* intragenic CGIs

**Fig 2:**
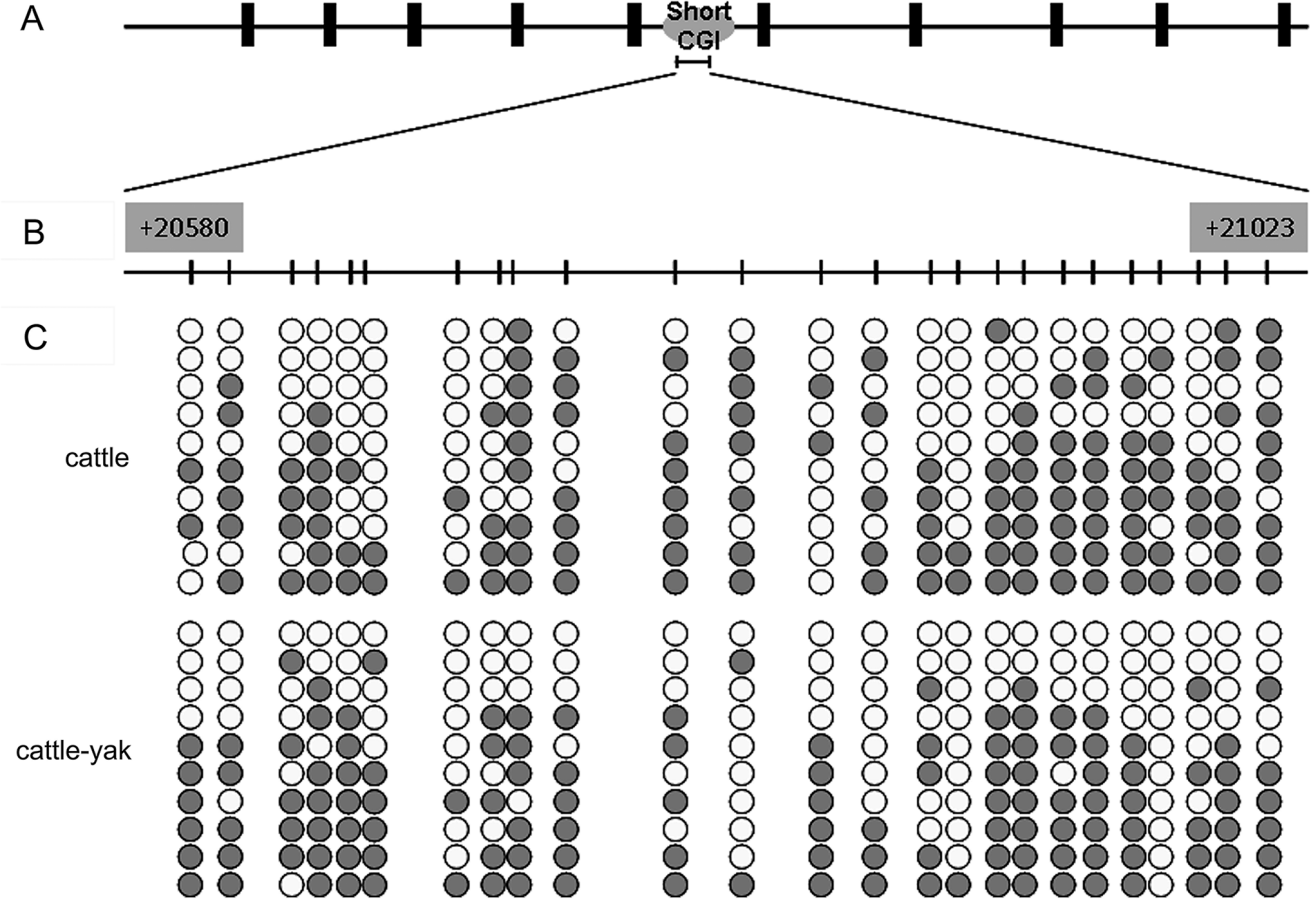
The methylation profile of the short CpG island in the b*Boule* gene body. (A) Schematic diagram of the short CGI within the b*Boule* gene body. (B) Schematic depiction of the CpG sites for methylation analysis. Nucleotide numbering is relative to +1 at the initiating ATG codon. The short vertical bars represent the CpG dinucleotides. (C) Methylation statuses of b*Boule* in testes of cattle and cattle-yak hybrids. Each line represents an individual bacterial clone that was sequenced. Open circles indicate unmethylated CpG sites. Black circles indicate methylated CpG sites.

Recent studies demonstrated that intragenic CGIs play an important role in regulating gene expression [37–39]. To assess the methylation status of short intragenic CGIs in cattle and cattle-yak testes, a 444 bp DNA fragment was amplified from the +20580/+21023 region of b*Boule* intron 5 with the P5 primers (**Fig 2A**). The amplified fragment contained 25 CpG sites (**Fig 2B**). Unlike the methylation of promoter CGIs, the b*Boule* short intragenic CGI methylation pattern was similar in cattle and cattle-yak testes (**Fig 2C**), and the difference between the methylation level of short intragenic CGI in cattle (52.0%, 130/250) and cattle-yak (51.6%, 129/250) was not significant (*P* > 0.05). These data indicate that methylation of short intragenic CGI is likely not associated with low b*Boule* expression in cattle-yak testes.

### Core promoter methylation level differed more in cattle and cattle-yak testes

**Fig 3:**
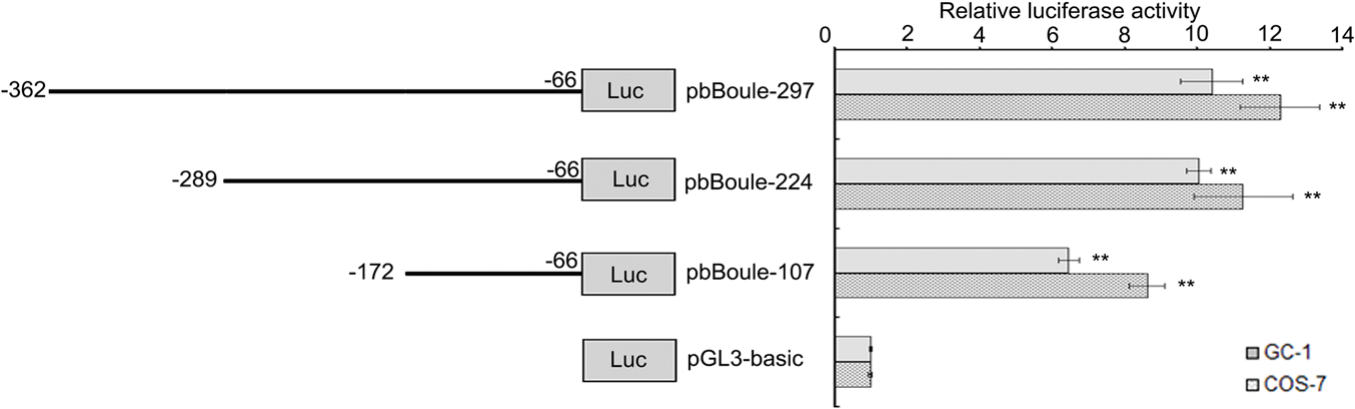
Identification of the core promoter in the b*Boule* gene. Left panel, functional deletion constructs of the b*Boule* 5' flanking region. Right panel, the luciferase activity of each deletion construct of the b*Boule* 5' flanking region. The deletion constructs were transiently transfected into GC-1 and COS-7 cell lines. Normalized luciferase activities are expressed as mean ± SEM of duplicates for a minimum of three experiments. All data were compared with the control group (pGL3-basic). ^**^ indicates a significant difference (P < 0.01).

To explore whether DNA methylation of the long CGI within the 5' flanking region contributes to the regulation of bBoule, we identified the core promoter region of b*Boule* by dual-luciferase reporter experiments. First, we predicted the 5' proximal flanking sequence from nt -408 to nt -158 as a potential core promoter region of b*Boule*. A series of deletion constructs (pbBoule-107, pbBoule-224, and pbBoule-297) were generated in the predicted promoter region (**Fig 3**), and GC-1 and COS-7 cells were transiently transfected. A luciferase activity analysis revealed that the pbBoule-107 construct is important for b*Boule* transcriptional activity, indicating that the basal promoter was located in the region from nt -172 to nt -66 (**Fig 3**). Further analysis showed that the core promoter of b*Boule* was located in the long CGI, and overlapped with the region examined in our methylation analysis. The core promoter included nine CpG sites, and the methylation level (45.56%, 41/90) of the core promoter region in the testes of cattle-yak was significantly higher than that of cattle (16.67%, 15/90) (*P* < 0.00001). However, among the 27 CpG sites outside the core promoter, the difference in methylation level between the testes of cattle-yak (8.52%, 23/270) and cattle (3.70%, 10/270) was small (*P* < 0.05). These data indicated that there was a greater difference in the methylation level between cattle and cattle-yak for the core promoter CGI than for the CGI outside the core promoter, and hypomethylation of core promoter CGI may be involved in low bBoule expression in cattle-yak testes.

### *In vitro* methylation represses b*Boule* promoter activity

**Fig 4:**
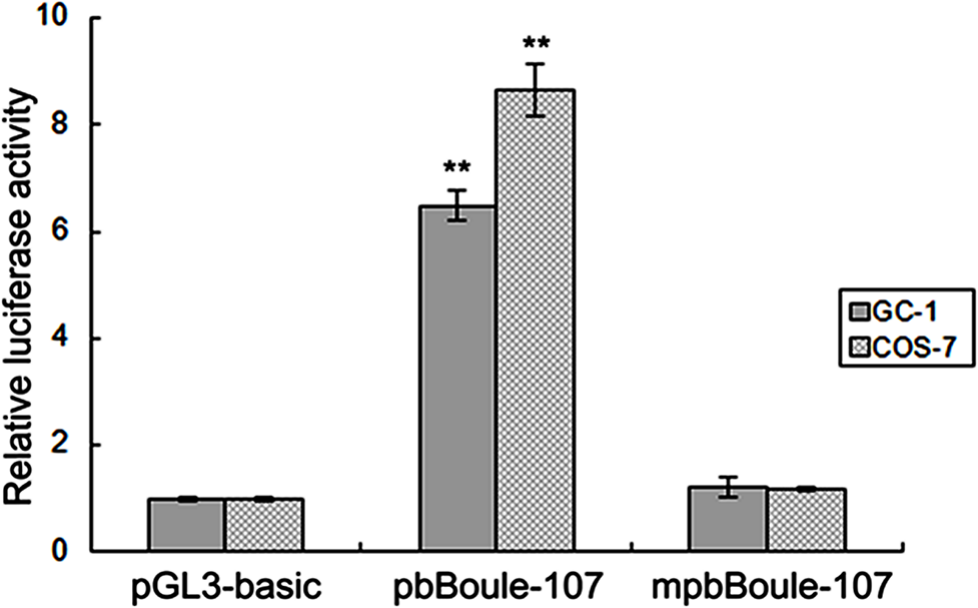
*In vitro* methylation assay of the b*Boule* promoter. The b*Boule* core promoter construct pbBoule-107 was treated with M.SssI methylase, and then methylated (mpbBoule-107) or unmethylated (pbBoule-107) plasmids were transiently transfected into GC-1 and COS-7 cell lines. Normalized luciferase activities are expressed as mean ± SEM of at least three independent experiments. The bar above the histogram indicates the SEM. ^**^ indicate a significant difference (P < 0.01).

To further determine where b*Boule* promoter activity was regulated by methylation of the core promoter, we performed an *in vitro* DNA methylation assay using the DNA methylase M.SssI. The core promoter pbBoule-107 construct was treated with M.SssI methylase, then the methylated (mpbBoule-107) or unmethylated plasmids (pbBoule-107) were transfected into GC-1 and COS-7 cell lines. Luciferase assays showed that the activity of the b*Boule* core promoter in both GC-1 and COS-7 cells decreased significantly after DNA methylase M.SssI treatment (all *P* < 0.01) (**Fig 4**), suggesting that promoter methylation is important in repressing b*Boule* transcriptional activity.

### Demethylation increases b*Boule* expression

**Fig 5:**
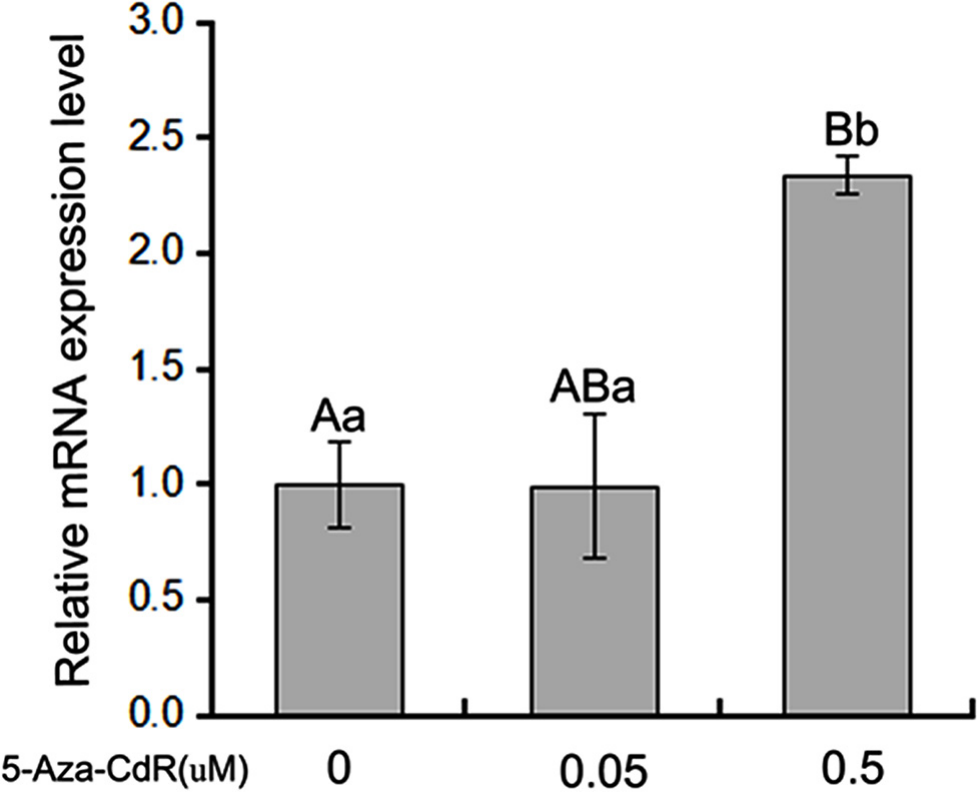
mRNA expression of b*Boule* in BMECs treated with 5-Aza-dC. mRNA expression was detected in treated cells but not in untreated cells by qRT-PCR. All experiments were performed three times. The bar above the histogram indicates the SEM. Different uppercase letters denote significant differences between different groups with a significance level of P < 0.01. Different lowercase letters denote significant differences between different groups with a significance level of P < 0.05.

To verify the association between promoter methylation and b*Boule* transcriptional activity, we treated BMECs that do not express bBoule with 5-Aza-dC, an inhibitor of DNA methyltransferase. b*Boule* mRNA expression was significantly higher in the 5-Aza-dC-treated group than the control group (**Fig 5**) (*P* < 0.01). Furthermore, the increased expression was dose-dependent (*P* < 0.05). These results further indicated that the transcription of b*Boule* was regulated by DNA methylation.

## Discussion

*Boule* is one of only two genes (*Boule* and *Nanos3*) that was directly shown to function in germ-cell development across diverse species including flies, worms, frogs, mice, and humans [5, 40]. *Nanos3* belongs to the *Nanos* gene family, and is expressed in the primordial germ cells of mammals; *Nanos3* knockout mice have smaller gonads and infertility in both male and female mice [40, 41]. *Boule* is a member of the *DAZ* family and is expressed in germ cells during the first meiotic division of mammalian spermatogenesis, and loss of function of mammalian Boule results in male-specific infertility [5, 42]. Our previous study found that b*Boule* is expressed at low levels in the testes of cattle-yak, a hybrid offspring of cattle and yaks, with male cattle-yak infertility caused by meiotic arrest [8, 33]. However, the epigenetic regulation mechanism of low b*Boule* expression is not known. DNA methylation is one of the most common epigenetic modifications in vertebrates; it regulates gene expression and thus affects gene function by influencing chromatin structure, DNA conformation, chromosome stability, and the interaction between DNA and proteins [37, 43–44]. In this study, we demonstrated a higher methylation level of the *bBoule* 5' region in cattle-yak testes with low b*Boule* expression and male infertility than in cattle with normal spermatogenesis (*P* < 0.01). Thus, methylation of the long CGI in the promoter may contribute to testicular b*Boule* transcription and male infertility. In fact, methylation in the promoter regions of many spermatogenic cell-specific genes is associated with male sterility, such as *PIWIL1* [35, 45–46], *PIWIL2* [46–47], *DAZL* [26, 28, 48], *SNRPN* [6, 49], *MEST* [6, 50], *VASA* [34, 51], and *MTHFR* [51–52]. Therefore, in the DAZ family, the methylation of two members, which exist in all mammals, *DAZL* and *Boule*, is associated with male sterility [28, 48], while the methylation of *DAZ*, another DAZ family member only found in primates, is not associated with male sterility [53].

In vertebrates, cytosine methylation is predominantly restricted to CpG dinucleotides and stably distributed across the genome, and regions with a high frequency of CpG sites are considered CGIs. CGIs are distributed throughout the genome, including in 5' promoter regions, gene bodies (coding regions and introns), 3' regions, and intergenic regions. In the past two decades, many experiments showed that CGI hypermethylation in 5' promoter regions represses gene transcription [34, 38, 54]. However, it was only recently discovered that CGI methylation in gene bodies is also distinctly involved in gene expression [37, 55–56]. Maunakea et al. [37] demonstrated a major role for intragenic methylation in regulating cell context-specific alternative promoters in gene bodies, and methylation of CGIs is more common in intragenic regions than in 5′ promoter regions in the human brain. A recent study showed that DNA methylation is not the key determinant in the regulation of most promoters in human HCT116 cells, but demethylation has a major effect on promoter-distal regulatory regions, uncovering intragenic enhancers within genes whose expression increases in the absence of DNA methylation [56]. This indicates that DNA methylation plays a distinct role in the silencing of regulatory elements within gene bodies. However, the methylation status of the short intragenic CGI in intron 5 of b*Boule* in the testis did not differ between cattle and cattle-yak. Similarly, methylation of a CGI in intron 1 of *GNA11* does not show a clear correlation with its decreased expression in human breast cancers [57]. Zhu et al. [58] reported that the methylation status of intragenic CpG islands-1 in *SHANK3* is not changed in brain tissues of patients with autism spectrum disorders. These observations suggested that the methylation level of intragenic CGI was not associated with low b*Boule* expression in the testes of cattle-yak hybrids or with male infertility.

In mammals, CGIs were found in or near approximately 40% of gene promoters [59]. Currently, studies of DNA methylation regulation of the expression of single genes mostly focus on methylation of CGIs in promoter regions, and hypermethylation generally inhibits promoter activity, whereas hypomethylation activates gene transcription [35, 52, 56, 60]. Here, we found that the difference in methylation level between the testicular tissue of cattle and cattle-yak hybrids was bigger for the core promoter CGIs than for those outside of the core promoter, indicating that high methylation of CpG sites in the core promoter was strongly associated with low b*Boule* expression in cattle-yak testes. The treatments with DNA methyltransferase (M.SssI) and the inhibitor of DNA methyltransferase (5-aza-dC) are the main direct *in vitro* methods to confirm that promoter DNA methylation regulates gene expression [61–65]. We further found that the activity of the b*Boule* core promoter decreased significantly after DNA methylase M.SssI treatment in GC-1 and COS-7 cells, while inhibition of DNA methylation with 5-aza-dC resulted in an approximately 2.5-fold induction of b*Boule* mRNA expression in BMECs. Our study provides strong support that DNA methylation inactivates the endogenous b*Boule* promoter, and exerts a negative effect on mRNA expression of b*Boule* in cattle-yak testes.

**Fig 6:**
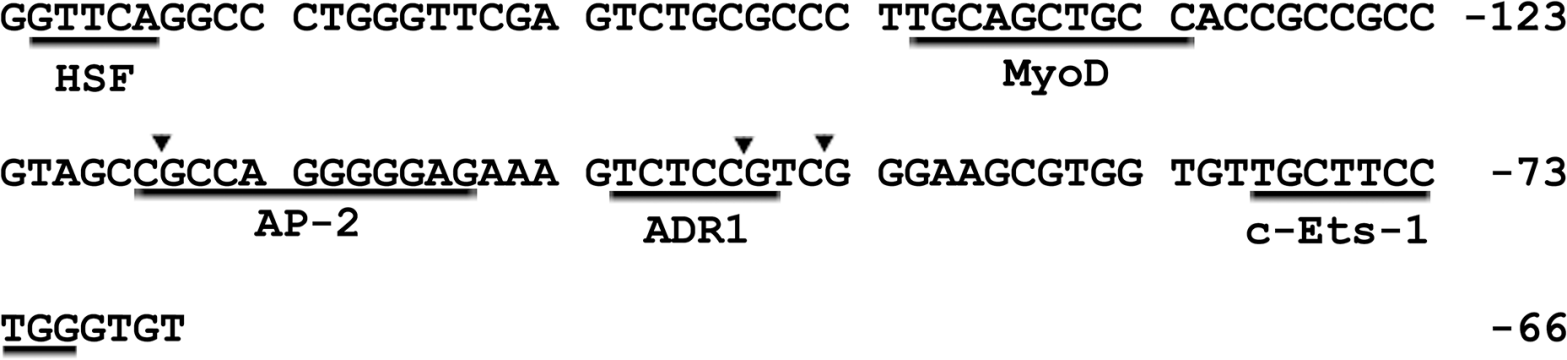
The predicated TFBS of differentially methylated CpG sites within the b*Boule* promoter. Arrows indicate differentially methylated CpG sites. The TFBS is underlined.

DNA promoter methylation could inhibit gene expression through direct interference with transcription factor binding to promoters, direct binding of specific transcriptional repressors, or alterations of the chromatin structure [35, 66–67]. To explore the molecular mechanism of DNA methylation inhibiting b*Boule* expression, we analyzed the methylation level of all CpG sites in the core promoter and found three differentially methylated CpG sites (-117CpG, -97CpG, and -94CpG). We next identified putative TFBS associated with the differentially methylated CpG sites using TFSEARCH v1.3 software (http://www.cbrc.jp/research/db/TFSEARCH.html), and found that -117CpG and -97CpG are located in the binding site for the transcription factors activator protein (AP)-2 and alcohol dehydrogenase gene regulator 1 (ADR1), respectively, while no known TFBS was predicted for the -94CpG region (**Fig 6**). AP-2 is a sequence-specific DNA-binding protein family including AP-2α, AP-2β, AP-2γ, AP-2δ, and AP-2ε, each of which binds to a GC-rich recognition sequence present in promoter and enhancer sequences, forming a vital link between *cis*-regulatory DNA elements and the general transcription machinery [68–70]. Bennett et al. [71] found that AP-2α expression is associated with target gene methylation and decreased expression in HNSCC cell lines, and demonstrated that AP-2α acts as a suppressor for certain “tumor suppressive” genes by targeting promoter methylation and/or deacetylation via HDAC recruitment. Adr1 is a transcription factor from *Saccharomyces cerevisiae* that belongs to the family of Cys2His2-type zinc finger proteins and regulates ADH2 expression through a 22 bp palindromic sequence [72–74]. However, there are no reports about the relationship between Adr1 and methylation of target genes in mammals. Therefore, hypermethylation of the AP-2 binding site (-117CpG site) in the b*Boule* promoter in cattle-yak testes probably causes reduced b*Boule* expression. Taken together, we speculate that methylation of the -117CpG site likely prevents AP-2 binding via disruption of its target sequence, which in turn hinders the recruitment of epigenetic factors, such as HDACs to the b*Boule* promoter, and results in b*Boule* repression; however, further experimental verification is needed.
